# Human rhinovirus infection induces local and systemic immunological tolerance in healthy volunteers

**DOI:** 10.1186/2197-425X-3-S1-A828

**Published:** 2015-10-01

**Authors:** RM Koch, M Kox, G Ferwerda, J Gerretsen, S ten Bruggencate, JG van der Hoeven, MI de Jonge, P Pickkers

**Affiliations:** Radboud University Medical Center, Intensive Care, Nijmegen, Netherlands; Radboud University Medical Center, Pediatrics, Nijmegen, Netherlands

## Introduction

A large proportion of ICU patients suffer from respiratory virus infections. This is often complicated by secondary infections, suggesting increased vulnerability in these patients. Recent work has shown that in bacterial sepsis, a immunosuppressive state called “immunoparalysis” accounts for this increased vulnerability. However, virus-induced immunoparalysis is largely unstudied. Human Rhinoviruses (HRVs) are the most frequent cause of the common cold. The “experimental cold model” is widely used to investigate the pathogenesis of HRV infection. However, the effects of repeated HRV exposure and thus possible development of virus-induced immunoparalysis have never been studied. Furthermore, although the virulent HRV-C can cause systemic and severe infections in both children and adults and the less virulent HRV-A strain can cause severe infections in immunocompromised patients as well, the HRV-A-induced systemic inflammatory and lower respiratory tract effects have never been studied in healthy subjects. Finally, it remains to be determined, whether serostatus alters the HRV-induced inflammatory response.

## Objectives

1. To investigate the effects of serostatus and repeated HRV exposure on the HRV-induced inflammatory response.
2. To assess HRV-induced systemic inflammatory and lower respiratory tract effects.

## Methods

In this randomized, double-blind, placebo-controlled study, 40 healthy, non-smoking, non-asthmatic male and female (1:1) subjects (seronegative: n = 22, seropositive: n = 18) were inoculated with HRV-16 (HRV-A) (n = 20) or placebo (n = 20). One week later, all subjects were inoculated with HRV-16. Nasal wash, blood samples and peak expiratory flow were obtained daily.

## Results

**Figure 1:**
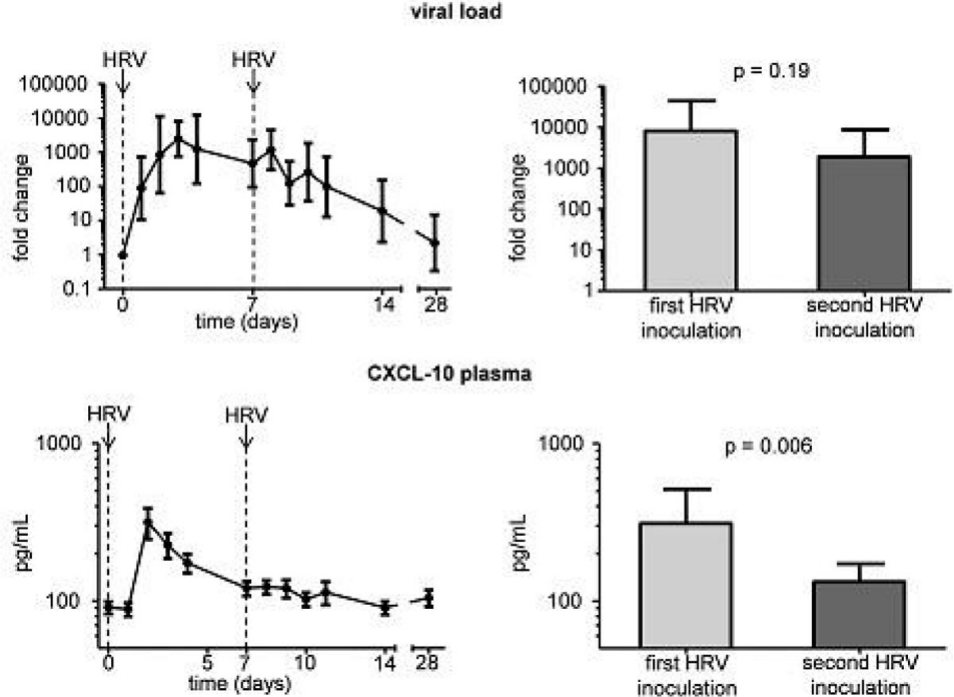
Figure 1

**Figure 2:**
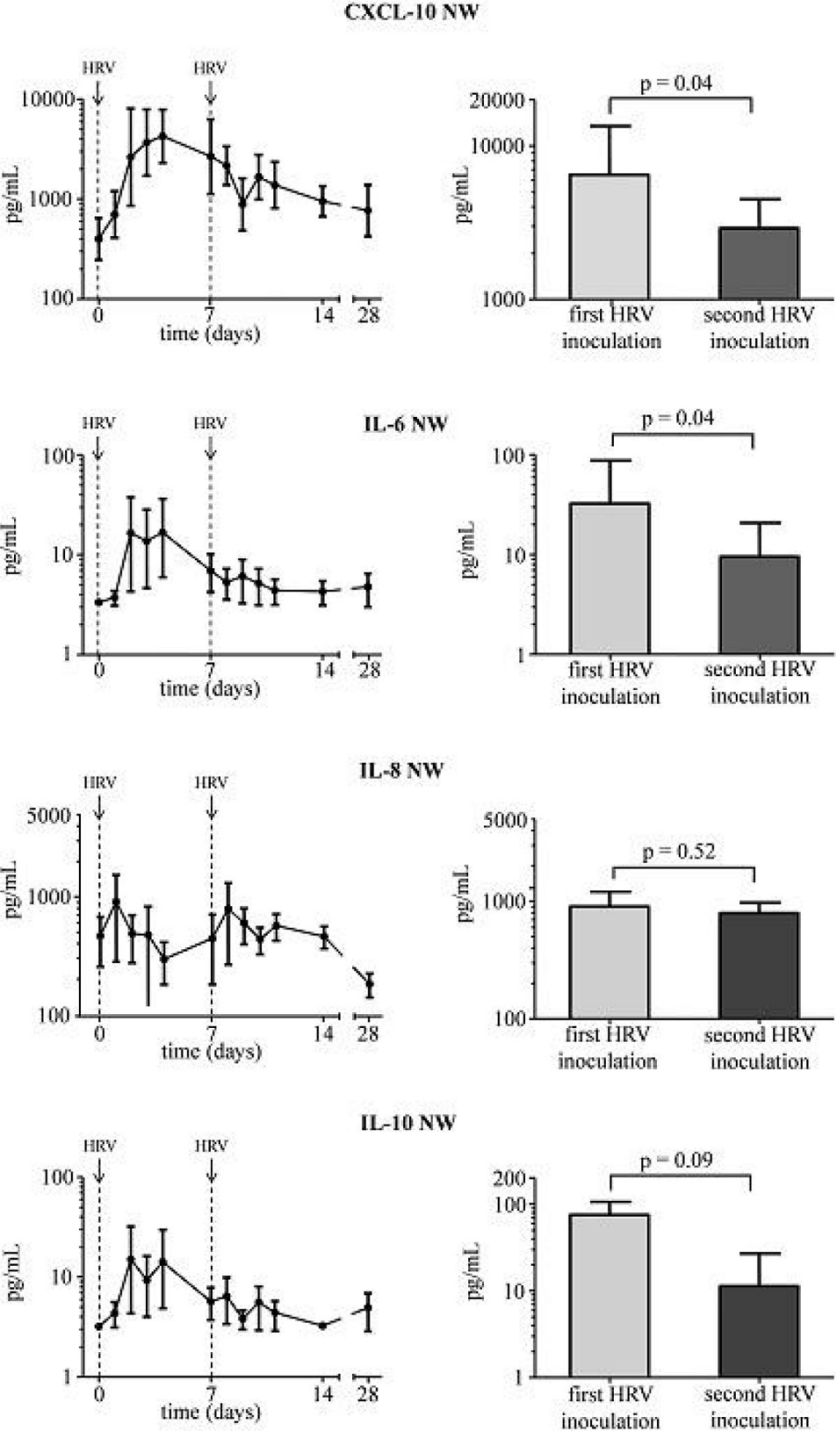
Figure 2

HRV-inoculation resulted in an infection rate of 82%. In seropositive subjects, HRV infection did not cause a local or systemic inflammatory response. In seronegative subjects, HRV infection resulted in increased levels of CXCL-10 (both in plasma and nasal wash), IL-6, IL-8, and IL-10 (in nasal wash). Despite similar viral load (Figure 1, upper panels), levels of CXCL-10 and IL-6 in nasal wash showed no increase but further declined upon the second HRV inoculation, and a similar trend was observed for IL-10 (Figure 2). Furthermore, the increase in plasma CXCL-10 levels observed after the first HRV inoculation was abolished upon the second HRV inoculation (Figure 1, lower panels). HRV infection did not affect peak expiratory flow.

## Conclusions

HRV infection does not result in an inflammatory response in seropositive subjects and exerts no effects on the lower respiratory tract. Furthermore, a second inoculation with HRV one week after the first results in a diminished local and systemic inflammatory response. This could be an explanation for the increased vulnerability towards secondary infections after respiratory virus infections in ICU patients.

## Grant Acknowledgment

This study was funded by an EFRO grant (2011-013287).

